# Less Is More: A Narrative Review of Deciding When Surgical Intervention Should Be Withheld

**DOI:** 10.7759/cureus.23285

**Published:** 2022-03-18

**Authors:** Annabel Stout, Jack Hamer, Tahlia Sharples, Farshad Tahmasebi

**Affiliations:** 1 Obstetrics and Gynaecology, Birmingham Women's Hospital, Birmingham Women’s and Children’s NHS Foundation Trust, Birmingham, GBR; 2 Obstetrics and Gynaecology, Walsall Manor Hospital, The Walsall Healthcare National Health Service (NHS) Trust, Walsall, GBR; 3 Obstetrics and Gynaecology, Gloucestershire Hospitals NHS Foundation Trust, Gloucester, GBR; 4 Obstetrics and Gynaecology, Russells Hall Hospital, The Dudley Group NHS Foundation Trust, Dudley, GBR

**Keywords:** patient-centred care, conservative management, surgery, obst gynec, multidisciplinary decision-making

## Abstract

Decision-making in surgery is one of the great unspoken challenges, an important but markedly challenging skill that takes a lifetime to master. The choice not to operate generally proves a significantly harder conclusion than opting for intervention. Our paper explores the influences which affect a clinician’s decision to operate or not. The challenging situation where patient and clinician disagree on proposed management is also explored. Implications on management are discussed, with constructive communication techniques offered, including the recent COVID-19 pandemic.

## Introduction and background

Decision-making in surgery is one of the great unspoken challenges, an important but markedly challenging skill that takes a lifetime to master. There are common situations where surgical intervention is not considered the superior management option; however, these decisions tend to be a lot harder to reach and communicate than the decision to operate.

Analyzing the risk versus the benefit of treatment is a core requirement in all fields of medicine, but decisions to withhold more extensive forms of care have generally been better mastered by our colleagues in other fields such as geriatrics and oncology, rather than by surgeons. The notion is important, with risks of futile, or worse inappropriately dangerous surgery being performed in poorly considered situations. This review explores the variety of patient and clinician determinants that may influence the decisions made whether to withhold surgical intervention or not. 

## Review

Factors influencing surgical decisions

Over recent times the paternalistic clinician approach to patient consultations has been largely rejected, leading to the desire for shared decision-making. By clinicians and patients working together during consultations, it is aimed to respect patient expectations and goals, whilst achieving the ability to integrate care plans that clinicians believe are within the patient's interest. A recent paper looking at shared decision-making regarding the management of mid-shaft clavicle fractures highlighted that 67.6% of patients would prefer to make a shared decision with their doctor [1]. This can be correlated with a recent review in 2020 whereby 54% of chosen papers that included patients and 75% of papers including surgical opinions both displayed a preference for shared decision-making [2]. Patient involvement within consultations will allow for increased patient understanding within one's own medical care, ensuring patients retain more information, appraise available options, or deliberate alternative management choices that afford the opportunity to make sensible decisions. It has also been shown to improve patient quality of life and satisfaction of the recommended treatment options, despite what the end outcome may be [3]. Surgeon involvement within shared decision-making allows for greater transparency on available management options, allowing clinicians to convey more than one option through the use of evidence-based preferences that center on patient values through appropriate education. 

Gunaratnam et al. identified five variables of shared decision-making: information (guidelines and evidence), medical condition (diagnosis, prognosis), institutional factors (resource availability), patient factors (age, personalities, co-morbidities), and surgeon factors (experience, peer support) [4]. Decision-making varied considerably amongst their cohort, as was demonstrated throughout evidence in this area. Morris et al. built further upon surgeon’s influences [5]. Externally, patients and relatives play a considerable role with the fear of feeling that nothing has been done to ameliorate their problem. Even if likely to be futile this can be too challenging for patients and relatives to bear. Ninety percent of study respondents cite these influential pressures [5]. An interesting notion regarding patients’ autonomy is considered. If a patient has received and understood full counseling, including relevant medical advice against operating, but still wishes for surgical intervention, does this become a patient’s autonomous decision to insist on surgical management? The concept of shared decision-making is universal within practice, however, respondents here suggest situations where a patient’s opinion may overrule doctors [5]. A challenging concept to consider given known surgical risks, forcing one to consider which opinion is ‘more important’. Where agreement cannot be met, to compare a clinician’s experience and knowledge with a patient’s will in the preferred management of their own body, and to then chose which opinion should be upheld is a significant ethical dilemma. Use of a second opinion in such cases is recommended.

A patient’s will in their own body management can be influenced by a variety of demographic factors, all of which require clinicians to provide forethought in ensuring adequate patient-provider communication is achieved. Sun et al. revealed how an increase in age is associated with greater proactivity in health outcomes [6]. However, lower education levels are positively associated with a poorer capacity for health decisions [6]. Promotion of patient's own health initiative should be one of the key forefronts for clinicians and requires care providers to tailor one’s consultation to specific patient needs. When deciding that surgical intervention may not be the preferable option, it is vital to modify one's language and idioms to each individual patient. This ensures the capacity to understand and increased patient engagement can be achieved. Broad assumptions of patients' understanding can be avoided with heedful communication tactics employed by clinicians through regular communication education.

It can be simple for clinicians to categorize patients by age and co-morbidity, making presumptions on what their prospective care should be, prior to patient discussion. Elderly patients with proposed high-risk procedures may be deemed unsuitable early on. However, if a patient is adamant they are willing to undertake the risk for the possible benefit to their lifestyle, the decision not to undergo active management may be made for them. Similarly, patients may unexpectedly refuse surgery, although a condition such as malignancy is life-threatening, a patient may not wish for surgery and prefer to continue their remaining time without the associated pain and immobility bought about by surgery. Presuming a patient's decision prior to the discussion is not an ethical approach for clinicians. 

Decisions may appear more challenging within high-risk procedures, and the notion of ‘life at all costs’, should not be the only case we consider [5]. Minor operations can still be associated with significant risks. In gynecological practice, patients will commonly request laparoscopic sterilization for permanent contraception. Whilst use of an intrauterine coil provides reduced risk and increased success, therefore is recourse to sterilization generally the best route? Many clinicians will recall cases where a severe complication has occurred in a perceived simple case and one should never feel entirely reassured they are immune from this.

The influence of medical decisions can not only be based upon relatives and patient influences, nor solely on the complexities of medical procedures, but upon a married influence of clinician experience and decision-making uncertainty. Anadan et al. examined responses from 158 surgeons relating to a variety of influences on surgical decision-making. Within this study, 91% of the surgeons agreed that their age and experience has a strong influence on the decisions made by them, while 87.2% of them felt that the institution from which they graduated influenced their decisions [7] This is also concurred by Gunaratnam et al. [4]. It is reasonable to believe that increased experience will allow clinicians to hold better judgment on which types of patients may, or may not be suitable for surgical intervention. Much of what is learned throughout surgeons' careers comes from assimilation of knowledge through repetition and learning from adverse outcomes, which in turn may provide surgeons with increased confidence when deciding not to operate. Influences due to where a surgeon previously graduated from may seem like a bizarre influential factor, however, dependent on the availability of surgical exposure, training, and familiarity of procedures throughout training, this may lead to those with less exposure becoming more hesitant to authorize decisions on whether or not to consider withholding a surgical intervention. 

Politi et al. studied patient and physician ‘anxiety to uncertainty’ and displayed a significant relationship between physician anxiety and patient outcome satisfaction [8]. Lawton et al. revealed an association between more experienced doctors making less risk-averse decisions, whilst feeling far more at ease with decisions of uncertainty [9]. The dyadic relationship between care providers and patients is key to ensuring a platform for patients to make entitled decisions about their own health is made. This is particularly important within the context of senior surgeons when the outcome of a non-surgical option may be deemed appropriate, albeit an uncertain option. Thus, it is key to managing patient health anxiety whilst conveying information that is perceived to be less ‘uncertain’. Experienced physicians may become more anxious regarding patient care, however in turn this can be translated across to patients with interpersonal communication and lengthened consultation times. The crux of shared decision-making is vital to sustaining patient-clinician relationships. It is therefore key for emerging surgeons to develop skills for coping with uncertainty early to warrant effective discussions with patients.

Internally, clinicians commonly site a feeling of need to ‘just do something’; rendering a management option entirely futile before trying it can be challenging [5]. As clinicians, active management of patients is ingrained within us from medical school; this is the practice we are used to. To go against this, ‘doing nothing’, is a skill that can be markedly harder to master [5]. Greater experience may improve confidence in assessment and resistance against pressure. The study proposes that objective assessment tools could be used in order to increase surgeons' confidence in advising against operative intervention. Albeit this is a skill that many clinicians would agree has been employed more frequently during the recent COVID-19 pandemic.

The recent pandemic has been a relevant influence on the surgical decisions made by both patients and clinicians alike. The pandemic has enabled us to foster skills we did not know we had. Guidance is available for all parties to make informed decisions albeit has been slow to disseminate. Consequently, clinicians have had to pivot their practices, prioritizing access to surgery to a level never previously required. Morris et al. stands with the use of laparoscopic surgery during the pandemic, as it provides improved patient outcomes with reduced risk to clinicians if sufficient protective equipment and modifications to operative standards are enforced, along with reduced admission duration [10] 

The paucity of evidence presented for clinicians to adhere to during the COVID-19 pandemic means that a proactive rather than reactive notion has been required by the medical community. Meeting the status quo for surgical procedures needs not to be the forte, but rather balanced with the championing of patient safety, whilst caring for patient fears and expectations. Vanni et al. revealed how influential patient fears of COVID-19 had upon their medical decisions, leading to increased refusal of operation consent or request for second opinion [11]. As previously mentioned, treatment refusal is an autonomous decision, however, decision-making on the basis of fear and uncertainty needs to be remedied by exploration of understanding and education through a clinician’s role. Whilst decisions for no surgical intervention should be vital in particular patient demographics, ensuring patients with surgically viable pathologies are appropriately guided is of paramount notability. 

Risk-benefit analysis and quality of surgical decisions

Sacks et al. found that assessment of risk versus benefit of surgery is highly predictive of the decision to operate, but the individual calculation of risk is highly variable [12]. They used computer-generated calculations to assess risk and found increased consistency in surgeons using these calculations versus those who did not. Surgeons using the tool calculated lower risk predictions, however, despite this, the decision to operate remained the same between cohorts [12]. Perhaps this signifies a reluctance of surgeons to go against their intuition? With years of training in decision-making, developing one's skills as seniority increases and awareness of the gravity of these judgments it is unsurprising surgeons would not be immediately willing to accept a computerized judgment as superior to their own. Work remains contradictory in this area, with studies concluding clinical judgment or prognostic tools more accurately estimate risk [13-15].

Despite the deliberation of employing computerized calculations for surgical evaluation, published evidence on ‘best case/worst case’ has displayed positive outcomes on surgical communication and patient involvement. Kruser et al. initiated a qualitative tool to support in-the-moment decision-making for life-threatening emergencies, helping clinicians to correctly narrate the patient path through each option [16]. This in turn provides nuanced, key clinical advice, whilst ensuring patients capture the understanding of their own health needs [16]. Study participants appreciated how the tool explained difficult situations in a clear and compatible way, which is particularly important when decisions to not perform a surgical intervention are discussed. Current gaps within surgical communication do not necessarily lie with the knowledge deficits clinicians have, but rather the delivery of such knowledge to patients. Computerized and qualitative tools enable clinicians to tailor their communication skills, ensuring patients become aware of the limitations of surgical interventions whilst thinking of the prospective outcomes in their own care. The consequence ensures a sustained and improved patient-provider relationship, allowing for unambiguous conversations regarding difficult topics.

Changing clinical practice

Formal guidelines are near impossible to formulate given the diversity of these cases. Given their lack of backing from published protocols, decisions may demonstrate more litigation concern. By virtue of such decisions needing to be individual, this welcomes more opinions on correct management, opening surgeons up to potential difficulties.

Where pressure from patients or relatives is present, or if a clinician is self-aware enough to note their feeling of attempting some form of help despite futility, which may sit more comfortably than ‘doing nothing’, balancing options is important. Setting out the situation clearly and gauging patient views is essential to good practice, presumptions should not be made nor mentioned, leading to a risk of coercion. Optimal communication skills play a huge role, where possible multiple reviews are likely to benefit. The use of guidelines, computerized tools, and qualitative communication tools may help to legitimize clinicians' decisions not to treat or operate.

As value-based care becomes increasingly important, cost implications also play a role in decision-making. Computerized assessments can include cost implications, and may aid the mental burden upon a surgeon suggesting a lack of surgical intervention. Such tools are not universally practical, however. Emergency cases, as well as intra-operative decision-making, pose testing situations with limited time to consider. If these tools were bought into regular use, doctors would need to develop decision-making both with and without computerized assistance to cover such challenges.

Surgical specialties emphasize technical skills over communication more often compared with our fellow medical counterparts. Despite this, it is evident that communication plays a far greater role through a patient surgical journey and strong communication emphasis can substantially improve a patient’s well-being. The paucity of research available on surgical patient communication highlights the deficit we all see within practice across multiple specialties. To rebuttal this, sufficient communication is one of the key standards within Good Surgical Practice by the Royal College of Surgeons of England, however employment in real-time practice is limited [17]. Structured and focused communication teaching, patient simulation sessions alongside mandatory attendances to communication courses should and need to be institutionalized within surgical training and have displayed good evidence for our medical specialty colleagues. 

## Conclusions

Assessing when not to operate is a challenge for a multitude of reasons, but maybe aided by the use of computerized assessment tools. These will never replace a human’s decision-making but may aid decisions for more challenging situations, and aid communication. Second opinions should be sought to aid difficult decisions. Education in this area would be greatly useful to surgeons and perhaps could be adapted from that received in some medical specialities. The optimum balance between clinicians' intuition, guidelines, and evidence-based computer analysis is difficult to decipher, and further research into this area is required. This review goes some way into demonstrating the need for development in this thought-provoking area.
